# Supplementary material for: Ethanol-guided behavior in Drosophila larvae
Source: Sci Rep. 2021 Jun 10;11:12307. doi: 10.1038/s41598-021-91677-3 (PMC8192949; doi:10.1038/s41598-021-91677-3)
Supplement: Supplementary file 1 — Supplementary Information 1. [file 41598_2021_91677_MOESM1_ESM.docx]

**Supplementary table legend**

**Supplementary Table 1: Statistical evaluation of the behavioral data.**  The table consists of nine sheets. These provide details on the title of the paper and the authors, show an index for the six figures, list the statistical tests used, and show all statistical analyses and their results for each behavioral experiment for all figures.

**Supplementary Table 2: Raw data of each behavioral experiments.** The table consists of six sheets; one sheet for each figure presented in the manuscript. Each sheet contains information about the genotype, the particular ethanol concentration used, and the individual preference value of each measurement. In some cases, additional information is provided on the test interval, specific feeding condition, time of larval development, and survival.
